# Creating an advance-care-planning decision aid for high-risk surgery: a qualitative study

**DOI:** 10.1186/1472-684X-13-32

**Published:** 2014-06-19

**Authors:** Anne LR Schuster, Rebecca A Aslakson, John FP Bridges

**Affiliations:** 1 Department of Health Policy and Management, The Johns Hopkins Bloomberg School of Public Health, Baltimore, MD, USA; 2 Department of Anesthesiology and Critical Care Medicine, The Johns Hopkins School of Medicine, Baltimore, MD, USA

**Keywords:** Advance care planning, Decision aids, Palliative care, Decision making, Preoperative care

## Abstract

**Background:**

High-risk surgery patients may lose decision-making capacity as a result of surgical complications. Advance care planning prior to surgery may be beneficial, but remains controversial and is hindered by a lack of appropriate decision aids. This study sought to examine stakeholders’ views on the appropriateness of using decision aids, in general, to support advance care planning among high-risk surgery populations and the design of such a decision aid.

**Methods:**

Key informants were recruited through purposive and snowball sampling. Semi-structured interviews were conducted by phone until data collected reached theoretical saturation. Key informants were asked to discuss their thoughts about advance care planning and interventions to support advance care planning, particularly for this population. Researchers took de-identified notes that were analyzed for emerging concordant, discordant, and recurrent themes using interpretative phenomenological analysis.

**Results:**

Key informants described the importance of initiating advance care planning preoperatively, despite potential challenges present in surgical settings. In general, decision aids were viewed as an appropriate approach to support advance care planning for this population. A recipe emerged from the data that outlines tools, ingredients, and tips for success that are needed to design an advance care planning decision aid for high-risk surgical settings.

**Conclusions:**

Stakeholders supported incorporating advance care planning in high-risk surgical settings and endorsed the appropriateness of using decision aids to do so. Findings will inform the next stages of developing the first advance care planning decision aid for high-risk surgery patients.

## Background

Recent studies report that since the late 1990s mortality rates have declined among patients who undergo high-risk surgery procedures in the United States, despite increases in the number of operations performed [1,2]. In spite of this improvement, there are inherent risks associated with any surgery, and complications, including death, may occur [3-6]. While high-risk surgeries performed by high volume surgeons and hospitals are associated with better patient outcomes [4,7], a subset of patients will still experience complications [3-7]. Facing such complications, it would be advantageous for patients preparing for high-risk surgery to engage in advance care planning—a process by which patients make preparations for medical decision making and engage in behaviors to guide medical decisions made on their behalf in the event that they cannot make their own decisions at a future time [8-11].

According to the Center for Disease Control (CDC) only 30% of people in the U.S. have made advance-care plans [12]. In situations where patients are asked to make tough decisions there is consensus that patients would benefit from decision support [13-15], which could include interventions facilitated either independent of or in conversation with someone such as a health care provider [13]. Decision aids are a specific type of decision support intervention and, as described by Stacey et al., they are tools grounded in scientific evidence that aim to prepare people for active, informed participation in decisions about their healthcare options [15]. Supplementing interactions with clinicians, decision aids for ACP have, among other findings, demonstrated effectiveness in increasing patients’ knowledge about treatment options, reducing decisional conflict, clarifying patients’ treatment preferences, and increasing completion of advance directives [16-23]. None, however, have been developed or evaluated for use in a high-risk surgical setting [24,25].

Evidence indicates that patients can face steep consequences for preoperatively expressing limitations to their post-operative life-sustaining care [26,27]. Research suggests that many surgeons negotiate with patients until there is consensus on acceptable post-operative treatments—a practice identified and named “surgical buy-in” [26,27]. If agreements cannot be reached, patients may be denied surgery, or surgeons may be unwilling to withdraw unwanted life-supporting treatment post-operatively [26,27].

Given the paucity of ACP decision aids for use in high-risk surgical settings [24,25] and evidence that ACP in surgical settings can be met with challenges [26,27], the purpose of this study was to examine the appropriateness of using decision aids to support ACP among high-risk surgery populations as well as to examine the design of such decision aids.

## Methods

### Study subjects

To gather a diversity of opinions, key informants in the U.S. and Europe were identified through purposive and snowball sampling on the basis of their expertise and professional involvement in: 1) surgical clinical care and/or research; 2) medical decision-making research 3) palliative clinical care and/or research; 4) end-of-life care policy making; and/or 5) patient advocacy. As described in a recently published study protocol, additional research will gather patient and patient-family perspectives [28]. Individuals identified as key informants were informed about the study and invited to participate via email. Upon agreement to participate, an interview was scheduled and a summary of the interview protocol was sent to participants. The interview protocol described the study’s purpose and the interview’s structure. It also outlined the consent to participate, potential harms of participating, and example questions to be discussed.

### Data collection

This qualitative research provided an opportunity to discuss ideas about ACP in general and components of ACP that may or may not specifically benefit high-risk surgery populations. For instance, researchers were curious about aspects of ACP such as the importance of naming a surrogate, identifying advance treatment preferences, and completing an advance directive. It also served to map out approaches to address ACP in this population. In particular, the study gathered key informants’ perspectives on instrument-based ACP decision aids–support mechanisms that are separate from but complementary to conversations with clinicians.

The primary endpoint for data collection was theoretical saturation [29]. Interviews followed a semi-structured interview approach [30], whereby researchers followed an interview guide but asked follow-up and clarifying questions about comments that surfaced during the conversation. Some example questions included:

•How would you define ACP?

•What are your thoughts about ACP in a high-risk surgical population?

•How would you describe decision aids and are you familiar with any that aim to facilitate ACP?

•What are the merits and drawbacks of existing ACP decision aids?

•What do you think about using decision aids for ACP in a high-risk surgical population?

Participants were further invited to contribute opinions that they deemed relevant to the conversation. Interviews were conducted by telephone in English; they were not recorded, but researchers took de-identified notes that were stored on password-protected computers. Notes were shared with key informants through a password protected website. Key informants could approve and/or modify the notes to improve accuracy prior to analysis or inclusion in the manuscript as supporting evidence.

### Analysis

After each interview the researchers conducted preliminary data analysis to identify key points that required further elaboration in future interviews. When researchers conducted the interview together, one team member merged the notes. Interpretative phenomenological analysis (IPA), a qualitative research methodology [30-32], was used to analyze the collected data. IPA is used in health research [33-35] and is a process by which to make meaning of individuals’ described, lived experiences.

This methodology follows a four-stage process to look for and connect emergent themes as well as to note themes that are discordant across the interviews. The process included reading and re-reading each set of notes to gain familiarity with the overall account and entailed:

1) creating a comprehensive commentary of the notes from each interview; 2) extrapolating and clustering themes for each interview; 3) connecting themes and identifying thematic clusters across all of the interviews; and 4) summarizing themes and providing supporting comments [30-32].

The following describes each of these four stages in more detail. Stage 1 produced a detailed commentary of the interview and involved describing, summarizing and paraphrasing the remarks from each interview as well as developing an interpretation of them (i.e., looking at the language used and the context of remarks or concerns). Stage 2 of the process used the comprehensive commentary to look for and cluster higher-level themes by reorganizing the data, reducing its volume, and identifying patterns. After completing the first and second stages for each interview, Stage 3 involved connecting themes and identifying thematic clusters across all of the interviews. To facilitate this process researchers wrote each theme on a separate piece of paper and placed them on a large table. Then the themes were grouped, reorganized, and relabeled to explore similarities, nesting, and discordance. Lastly, Stage 4 produced a summation of the themes and clarified supporting comments. One analyst (ALRS) conducted stages 1 and 2 independently for each interview, and then the other researchers reviewed this work in order to assess the interpretation’s integrity to the data. Two researchers (ALRS, JFPB) jointly conducted stages 3 and 4 and worked to reach consensus.

### Ethics

All participants were informed about the study and its potential risks and benefits. Participation in the study was voluntary and respondents were not reimbursed for participation. Techniques were used to protect participant’s anonymity and confidentiality. The study was deemed exempt (#NA-00088616) from human subject**’**s consideration from the Johns Hopkins School of Medicine Institutional Review Board.

## Results

Among the twenty-six individuals contacted for interviews, four did not reply and twenty-two (85%) agreed to be interviewed. Subsequently, twenty-two in-depth interviews were conducted with leading clinical, research, policy, and patient-advocacy key informants from the U.S. (n = 21) and Europe (n = 1) whose work pertained to surgery (n = 7), palliative care (n = 12), and/or medical decision-making (n = 3). Sections that follow discuss the themes that emerged pertaining to the appropriateness and design of a decision aid for ACP in a high-risk surgery population.

Key informants discussed the need to better prepare for and handle situations in which patients cannot advocate for themselves. As one key informant put it,

Clinicians need to learn to ask what is important to the patient and to make it a priority, rather than an icebreaker. It saves time, it saves money, and it saves suffering. This is fundamental to clinical medicine.

*—* A policy-maker

Stakeholders voiced support for ACP when patients prepare for any serious procedure where mortality and major morbidity are of concern. As one key informant stated, *“These are issues to be discussed before patients reach the cliff – I think anyone diagnosed with a life limiting condition should have ACP started right from the start. It should not be an add on at some later time.*” It was suggested that surgery could be interpreted as a trigger point for initiating ACP because it is a defined moment in which patients will face uncertain outcomes. In particular, a respondent observed that “ACP *in surgical settings could be helpful because surgery tends to be the front door for lots of things that come in to the institution,”* and another noted that *“As a whole ACP in surgery has value but depends on the patient and procedure. Thinking beyond just death but towards instances where major morbidity is a concern warrants ACP.”*

Therefore, for non-emergent surgeries, patients have an ideal opportunity to make preparations for the potential event of not being able to make their own decisions, and thereby could prevent unnecessary family burden in moments of crisis. One respondent thought that there could be a greater readiness to address ACP with patients preparing for high-risk operations within certain sub-specialties of surgery, especially where there was a high probability of the patient being limited in some way after the surgery.

A few participants suggested that there be ways to alert the patient to initiate ACP discussions before surgery. Decision aids were viewed as a way to help empower patients and prompt such discussions, and one stakeholder specifically stated, *“I’m hungry for patient decision aids & tools that prepare patients for the conversation.”* Moreover, decision aids were seen as one approach to ease the burden on patients, families, and the health care system. The following comment helps illustrate this point: *“If decision aids would work optimally, they would make the process more comfortable for patients and families and more efficient for health care providers”.*

Despite recommendations that ACP be part of pre-surgery preparations, key informants discussed potential challenges to doing so. While some challenges addressed facets such as a lack of time and physician reimbursement schedules, significant difficulties specific to a surgical setting were also identified. A notable barrier to introducing ACP was an observed and particular reluctance to acknowledge or discuss death within the field of surgery. One stakeholder reflected on this and said:

Within all the cultures of medicine, surgery is the most death defying of all. It deals with death all the time, yet people don’t want to talk about it. This creates a disconnect.

– A clinician

Stakeholders referenced the practice of surgical buy-in as a barrier to ACP. One key informant who presented this in the form of a discussion between a patient and a surgeon, remarked that “*The surgeon might say*, *‘If we do X, we may need to do Y, Z, and A afterwards. If you are not willing to do this, then perhaps I am not the right person to do this or you may not want to proceed with this operation.’”* Familiar with these kinds of consequences, a few key informants cautioned against encouraging patients to complete advance directives as a form of ACP before surgery, as they could lead to a patient being denied surgery.

### Recipe

Respondents described, in essence, a recipe for designing a decision aid–they described the recipe’s tools, ingredients, and tips for success. For each part of the recipe there were three supporting elements. Table 1 outlines these three parts of the recipe and their supporting elements, in addition to offering representative comments. Each of these categories will be described in detail below.

**Table 1:** Recipe categories, supporting elements, and representative key informant comments

**Recipe category**	**Supporting elements**	**Representative comments**
Tools	Patient needs	“Very few decision aids have embraced the consumer’s needs and frame of reference.”
		“Focus groups help define content. Do interviews with patients and caregivers to hear ‘what freaks them out.’”
		“It’s not realistic that everyone will have the conversation. You’ve got to meet people where they are.”
	Surgeon needs	“A sick patient, surgeon has info, and their life is in surgeon’s hands, and many people will do anything surgeon says.”
		“I'm worrying about the degree to which a surgeon can extinguish or redirect something that he or she finds uncomfortable.”
		“Hard for the provider to divorce themselves from being the agent of a potential intervention.”
	Setting impact	“Settings such as using aids in a community vs. clinic, urban vs. rural are important to look at.”
		“These are discussions that should be at the dinner table, not in the ICU.”
		“There is an impression that DNR means do not treat – particularly in surgical world because surgery is very invasive.”
Ingredients	Current aids	“Many aids are not successful in overcoming the barriers in discussing end-of-life care such as thinking about death.”
		“Traditional decision aids are very long and no one likes to talk about death for very long.”
		“Videos require a remarkable amount of critical scrutiny in regards of bias and appropriate pilot testing with stakeholders.”
	New content	“The absolute most important piece of paper is a medical power of attorney. This is the person who is willing to speak up and say ‘I know X and she would want . . .’”
		“Decision aids open up dialogue and a communicative environment where one can express things.”
		“Any aids that get people to know and self reflect, communicate between patient and health care provider – those are good things.”
	Patients’ values	“To me it is a process where you develop a deeper understanding of where you are. It is thinking about your preferences and needs.”
		“Advance care planning should be a process to prepare for making potential decisions. This is more useful than considering hypothetical situations and making treatment choices.”
		“Advance care planning is not just whether you want a particular treatment but identifying what goals and values are important and aligning treatments with them.”
Tips for success	Keep it simple	“Is to the point but leads to something that you can measure down the line and honors choices, so people can get the care they want at the end of life.”
		“You need an intervention that is short and sweet. For instance, is short enough to watch it there with the surgeon.”
		“Spectrum of aids that swings from way too general to incredible layers of micromanaging.”
	Make it adaptable	“Functionality, tailoring of information are good. Will get constant feedback and have iterative changes over time.”
		“Describing CPR in words may be difficult for people with low health literacy to understand. Video testimonials gets around a lack in health literacy.”
		“Huge issue around health literacy and how to adapt aids to patients and families with low health literacy.”
	Show its effectiveness	“The gold standard question to ask is, do these decision aids lead to better patient outcomes?”
		“Would encourage that measurement of effectiveness be highlighted a lot, but you have to measure something that matters to people who are answering the questions.”
		“Missing a lot of data on implementation. If something works in a study on effectiveness, how do you implement it, achieve a desired outcome, and sustain it?”

### Tools

As tools for building an ACP decision aid for high-risk surgery, the following three sections elaborate on the importance of engaging with patients and surgeons as well as the importance of gauging the setting’s impact on ACP.

#### Engage with patients

Key informants stressed the need to design a decision aid that accounted for patients’ readiness to participate in ACP. This was deemed to be particularly relevant for patients approaching high-risk surgery because as several key informants noted, high-risk surgery patients are often hoping for the very best outcome, which may affect their readiness to initiate ACP. As one respondent said, *“Surgery patients are really shooting for a cure and they’re not interested in talking about what they want if things don’t go well.”* As such, a stakeholder expressed that a well-designed decision aid ought to *“consider stages of behavior change and get people from pre-contemplation or contemplation to activation.”*

The stakeholders advocated for what they termed “patient-centered decision aids” and felt that patient participation would be vital to developing successful aids. A participant stated, *“We don’t know enough about what the patient thinks and what the patient values at the time of a major surgical decision.”* To learn more about this population’s specific needs, some stakeholders suggested that focus groups with patients who have the illness of interest could be used as both a starting point and feedback loop to gather input. These patients could help provide insight on the decisions they encountered throughout treatment and could identify information or support that could have aided their decision-making. Furthermore, focus groups were viewed as a useful way to refine the content and design of a decision aid undergoing development.

#### Engage with surgeons

Key informants described the importance of understanding surgeons’ roles and accounting for them when designing a decision aid for ACP. Namely it was important to recognize that surgeons have not been eager to integrate palliative care, which can include ACP, into their work. As a respondent explained, *“Surgeons have been among the last to adopt palliative care. They can see it coming up because of a failing on their part or because they see it as outside their domain.”* In part, this could stem from what one key informant named ‘the great man syndrome,’ whereby the surgeon takes on the responsibility of saving a patient’s life*.* Similarly, a few speculated about the impact of 30-day outcomes reporting; this practice may bring about a surgeon’s hesitancy to work with a patient who expresses limits on life-sustaining treatment.

These issues point to the power dynamics that exist in the patient-physician relationship. One key informant remarked, *“A lot of power differentials exist. There’s a sick patient where a surgeon has info, and a patient’s life is in the surgeon’s hands. Many patient’s will do anything a surgeon says.”* Recognizing this dynamic, some respondents advocated for training surgeons better about how to have end-of-life conversations. Other key informants held that support mechanisms such as decision aids could be put in place as part of a collaborative model.

#### Gauge the setting

Key informants further described the impact that setting could play on facilitating ACP. Consequently, it was suggested that some settings would be more conducive. Hospitals and Intensive Care Units were especially viewed as less than ideal settings for these conversations, when citing the stressors of hospital stays and the pressures to make serious decisions under tight timelines. Considering these factors, a few respondents specifically hoped to see ACP conversations occur at patients’ homes around the dinner table. Also, decision aids in the form of a workbook were seen by some as being more amenable to home use because they would not require patients to have access to a computer or internet to complete them.

### Ingredients

Stakeholders had strong opinions about the essential ingredients of a decision aid, which were based on their own familiarity with or experience using existing ACP decision aids. Their opinions were also drawn from their observations of what is helpful in situations where a patient has lost their decision-making capacity. The next three sections describe these findings.

#### Shift focus from end-of-life

Respondents commonly held that a barrier to ACP was an overemphasis on dying. As one stakeholder stated, “*People are inherently and psychologically structured so that they don’t like thinking about death. It’s reinforced by our death defying culture.*” It was noted by another key informant that an emphasis on death and dying could be explicit or implicit in the way we talk about the issues: *“When we use the terms advance care, advance directive, or ‘advance anything,’ people automatically equate that with death and dying.”* Considering this barrier, some respondents observed that many existing ACP decision aids focus heavily on dying. A key informant expressed that besides a recently developed website by Sudore and colleagues [8], “*Everything else is the same old same old”.* In the opinion of key informants, this emphasis shuts down conversation.

#### Elicit patient values and goals

Key informants shared the opinion that ACP decision aids, particularly for patients approaching surgery, should focus on what patients enjoy about life. Often this was framed as a quality of life discussion. One stakeholder succinctly and enthusiastically recommended that decision aids should *“…pull out of the end-of-life rut and get into the quality of life groove.”* Another stakeholder elaborated that quality of life is a foundational component of ACP decision aids, by stating that, *“In the ideal world, decision aids would focus on helping people articulate what is important to them in terms of quality of life. That could drive everything that follows.”* There was consensus that instead of asking patients to predetermine their treatment preferences, patients should receive goal-directed care where treatment decisions flow from patients’ expressed goals and values.

#### Prompt naming a surrogate and having discussions

The key informants described certain elements that are vital to ACP. They commonly said that naming a surrogate decision-maker was a highly valuable outcome of ACP, particularly for patients approaching surgery. The following quote reflects this opinion: *“The main issue in surgical settings is to get people to think about what if things went bad. Getting them to name a surrogate and to talk to the person before having surgery would be a useful thing.”* As such, it would be important to include content that instructed patients to choose a surrogate and informed them about ways to evaluate whether someone would make an appropriate decision-maker. Moreover, respondents felt that effective decision aids would also facilitate conversations between a patient, their surrogate, and their health care providers.

### Tips for success

Stakeholders identified a number of factors associated with the likely implementation of a decision aid, and as discussed below, emphasized designing a decision aid that can be integrated in a clinical setting.

#### Keep it simple

Simplicity was identified as a vital criterion for a decision aid. A key informant remarked, *“We need to come up with pragmatic interventions that address the practical reality of patients who are facing these decisions.”* Many key informants thought that decision aids were often too cumbersome or lengthy to realistically expect patients to complete them: *“The vast majority of patients do not have the energy or interest to complete a 50, 20, or even 10-page booklet full of questions.”* To make decision aids user-friendly and to increase their chances of being adopted, respondents recommended balancing the accessibility of information with user’s attention span.

#### Make it adaptable

According to stakeholders, decision aids need to be adaptable over time and across users. Stakeholders noted that decision aids require iterative changes for improvement. One respondent recommended using websites specifically because of the ease of revising a website versus the energy and expense of reproducing materials in other mediums, such as paper or video. Another way in which key informants discussed adaptability was in terms of responding to users. In particular, patients’ health literacy levels were discussed. Some key informants suggested that decision aids be designed at a fifth-grade reading level, whereas others noted that a more useful and long lasting aid would be able to speak to numerous user groups while also adapting to those with lower health literacy. Lastly, stakeholders believed that decision aids ought to respond to users’ unique needs. The ability to tailor a decision aid would ensure meeting the needs of patients at varying levels of readiness to engage ACP.

#### Show its effectiveness

Respondents identified the need to inform the development of decision aids with relevant theory as one indicator of effectiveness. A representative comment from one participant was,

There are so many tools out there, but they need to be based on evidence. Very few teams bring in the health communication experts and bring in the adult learning knowledge base—many tools don’t understand the process of how people come to make a decision.

—A policy maker

Further, stakeholders observed a need to evaluate and subsequently, if appropriate, promote a decision aids’ effectiveness. They wanted decision aids to be linked to patient outcomes such as decreasing the number of patients who die in the ICU who clearly had a diagnosis that the ICU wouldn’t help. One stakeholder commented, *“I feel good when I use decision aids but I don’t know if they translate into any changes in medical care.”*

## Discussion

Support for ACP in high-risk surgical settings is controversial. Our findings acknowledge the challenges and hesitancies to implementing ACP in these settings. However, among our key informant interviewees, there was support for patients approaching high-risk surgery to initiate ACP focused on identifying patient values and goals, naming a surrogate, and having discussions with surrogates, family and health care providers. Emphasis on these outcomes resonate with the recent reconceptualization of ACP; one that emphasizes preparations for making medical decisions in the face of uncertainty [8-11].

A decision aid was also conceived as an appropriate tool for ACP within a high-risk surgery setting. The herein identified ingredients of a decision aid for ACP among a high-risk surgical population respond to an identified need to establish decision aids’ essential ingredients [15]. Additionally, this decision aid would need to tailor content to different users’ needs. Patient engagement via focus groups from development through to evaluation was identified as a vital component to the successful development of such an ACP decision aid. This adheres to a development process for web-based decision aids described by Elwyn et al. [36]. It was clear that input from surgeons would be equally important for a decision aid to be accepted within high-risk surgical settings. Without gaining support and incorporating surgeons’ perspectives it seems unlikely for a decision aid to be readily adopted.

There are some limitations of this study. First, the stakeholder sample may be relevant to the support this study gathered for developing and using ACP decision aids in surgical settings. A greater diversity of opinions may have been generated if our sample inclusion criteria had been broader or if we had interviewed more stakeholders from outside the U.S. where palliative care can be more deeply integrated. Second, the interviews were not recorded and the comments included here are taken from the interview notes. While not precise quotations, respondents were given the opportunity to confirm that we had captured their thoughts accurately and, if not, to adjust the notes we took to better reflect their perspectives. Third, we were not able to evaluate key informants’ lived experience of decision aids for ACP in a surgical population since none exist. Gathering insights on aids that are currently available, however, offered the most suitable approximation. When ACP decision aids for surgical settings are developed, IPA could be applied to specifically understand key informants’ experiences using them. Finally, given the nature of the research, the emergent themes are based on researchers’ interpretation of respondents’ comments and may be influenced by our own frame of reference and preconceptions. However, several standard qualitative research techniques were used to mitigate this.

The expressed interest in implementation aligns with others efforts to see shared decision-making research translated into practice [37,38]. Demonstrating a decision aids’ simplicity, adaptability, and effectiveness seem to address some previously identified necessary conditions for implementing them in clinical practice such as being high quality, meeting the needs of the population, and employing effective systems for delivery [15]. This necessitates evaluating new decision aids for their effectiveness. A central aspect to implementation will be how decision aids fit into established clinical and organizational systems. Here again, seeking surgeon support and securing their willingness to use ACP decision aids will be essential to successful implementation. As such it is crucial to implementation that a decision aid not unintentionally shut out the surgeon but rather truly promote shared decision-making.

## Conclusion

This study makes an important contribution by identifying a clinical need, demonstrating support for high-risk surgery ACP, and outlining pertinent components of an ACP decision aid for this population. A decision aid that emphasizes quality of life, elicits patient values, helps identify a surrogate decision-maker, and prompts discussions between a patient and their surrogate was deemed meaningful. These findings set the stage for future research to engage with patients and surgeons about developing an ACP decision aid for use in high-risk surgical settings.

## Competing interests

The authors declare they have no competing interests.

## Authors’ contributions

RA conceived of the study. RA, AS, and JB participated in the design of and development of the interview protocol. RA and AS coordinated and conducted the interviews. AS and JB carried out the data analysis and drafted the manuscript. All authors read and approved the final manuscript.

## Pre-publication history

The pre-publication history for this paper can be accessed here:

http://www.biomedcentral.com/1472-684X/13/32/prepub
